# Blue light emission from the heterostructured ZnO/InGaN/GaN

**DOI:** 10.1186/1556-276X-8-99

**Published:** 2013-02-22

**Authors:** Ti Wang, Hao Wu, Zheng Wang, Chao Chen, Chang Liu

**Affiliations:** 1 Key Laboratory of Artificial Micro- and Nano-structures of Ministry of Education, School of Physics and Technology, Wuhan University, Wuhan, 430072, People's Republic of China

**Keywords:** ZnO/InGaN/GaN heterostructures, Atomic layer deposition, Electroluminescence, 77.55.hf, 73.63.Bd, 73.40.Kp

## Abstract

ZnO/InGaN/GaN heterostructured light-emitting diodes (LEDs) were fabricated by molecular beam epitaxy and atomic layer deposition. InGaN films consisted of an Mg-doped InGaN layer, an undoped InGaN layer, and a Si-doped InGaN layer. Current-voltage characteristic of the heterojunction indicated a diode-like rectification behavior. The electroluminescence spectra under forward biases presented a blue emission accompanied by a broad peak centered at 600 nm. With appropriate emission intensity ratio, the heterostructured LEDs had potential application in white LEDs. Moreover, a UV emission and an emission peak centered at 560 nm were observed under reverse bias.

## Background

Nowadays, white light-emitting diodes (WLEDs) have attracted significant interest for solid-state illumination due to their low power consumption, long operating time, and environmental benefits [1-3]. Hence, WLEDs are the most promising alternatives to replace conventional light sources, such as backlighting, interior lamps, and general lightings [4]. Currently, the prevailing method is to use a blue LED coated with a yellow-emitting phosphor. However, during a long period of optical pumping, the degradation of the phosphor would decline the output efficiency of the WLEDs. Another way to obtain white light is to mix the emissions from different light sources [5]. In particular, InGaN with a continuously variable bandgap from 0.7 to 3.4 eV has attracted considerable interest, and thus, InGaN/GaN WLEDs are regarded as the most promising solid-state lighting device which can work in the whole visible and part of the near UV spectral regions [6]. Some groups have fabricated dichromatic InGaN-based WLEDs [7]. However, compared with WLEDs with a mixture of blue, green and red emissions, they had lower color rendering index.

With a direct wide bandgap of 3.37 eV and high exciton binding energy of 60 meV, ZnO is considered as one of the best electroluminescent materials. However, herein lays an obstacle of ZnO homojunction diodes, which is p-type; it is a problem in obtaining high-quality and stable p-ZnO films. Although some p-n homojunction ZnO LEDs have been reported, their electroluminescence (EL) intensities were very weak [8-10]. As an alternative approach, heterostructured LEDs have been fabricated on top of a variety of p-type substrates, such as SrCu_2_O_2_[11], Si [12], and GaN [13]. With the advantages of InGaN and ZnO, it is significant to fabricate ZnO/InGaN/GaN heterojunctions with blue, green, and red emissions to obtain white light.

In this work, we report the fabrication of ZnO/InGaN/GaN heterostructured LEDs. The EL spectra under forward biases presented a blue emission accompanied by a broad peak centered at 600 nm. With appropriate emission intensity ratio, heterostructured LEDs have potential application in WLEDs. Moreover, a UV emission and an emission peak centered at 560 nm were observed under reverse bias.

## Methods

**Figure 1:**
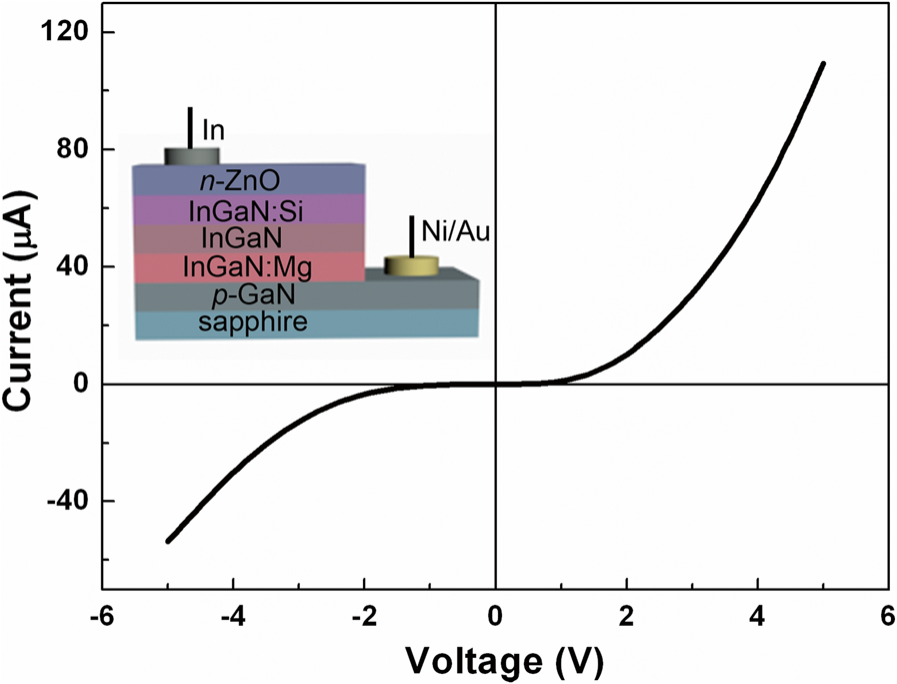
***I*-*V *curve of ZnO/InGaN/GaN heterostructure.** Inset shows the sketch map of the structure.

There were two steps to fabricate the ZnO/InGaN/GaN LEDs (inset of Figure 1). Firstly, InGaN films were deposited on commercially available (0001) p-GaN wafers on sapphire by radiofrequency plasma-assisted molecular beam epitaxy (SVTA35-V-2, SVT Associates Inc., Eden Prairie, MN, USA). A 7-N (99.99999%) Ga and 6-N (99.9999%) In were used as source materials. Nitrogen (6 N) was further purified through a gas purifier and then introduced into a plasma generator. The InGaN film consisted of a 150-nm Mg-doped InGaN layer, a 200-nm intrinsic InGaN layer, and a 400-nmSi-doped InGaN layer. Secondly, ZnO films were deposited on the InGaN films by atomic layer deposition (TSF-200, Beneq Oy, Vantaa, Finland). The detailed experimental method can be found in our previous work [14]. In this work, 4,000 cycles were performed, and the thickness of ZnO films was about 600 nm. In order to demonstrate the rectifying behavior that originated from the heterojunction, Ni/Au and In were fabricated as the p-type and n-type contact electrodes, respectively.

## Results and discussion

The photoluminescence (PL, HORIBA LabRAM HR800, HORIBA Jobin Yvon S.A.S., Longjumeau, Cedex, France) measurements were conducted at room temperature in the wavelength range of 350 to 700 nm to analyze the optical properties of n-ZnO films, InGaN films, and p-GaN substrates. In order to assess the performance of the heterostructured LEDs, current-voltage (*I*-*V*) and EL measurements were carried out at room temperature. The rectifying behavior with a turn-on voltage of about 2 V is observed in the *I*-*V* curve (Figure 1).

**Figure 2:**
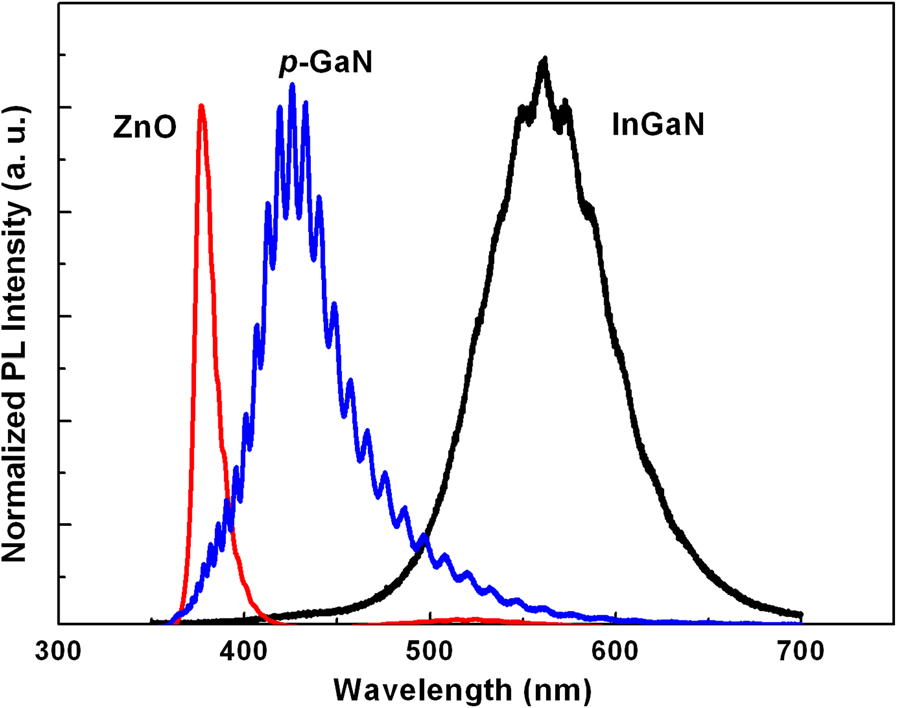
Room-temperature PL spectra of ZnO, InGaN, and GaN.

The room-temperature PL spectra of the ZnO, InGaN, and GaN layers are presented in Figure 2. As shown, the PL spectrum of p-GaN was dominated by a broad peak centered at about 430 nm, which can be attributable to the transmission from the conduction band and/or shallow donors to the Mg acceptor doping level [15]. Fringes were observed in the spectrum on account of the interference between GaN/air and sapphire/GaN interfaces [16]. The spectrum of InGaN:Si was dominated by a peak centered at about 560 nm. Because the total thickness of the intrinsic InGaN film and the Si-doped InGaN film was about 600 nm, the influence of Mg doping in InGaN cannot be observed from the PL spectrum. The spectrum of ZnO displayed a dominant sharp near-band-edge emission at 380 nm, and deep-level emission at around 520 nm was not observed. Deep-level emission has been reported to be caused by oxygen vacancies. Therefore, it indicated few oxygen vacancies existing in the ZnO films [14].

**Figure 3:**
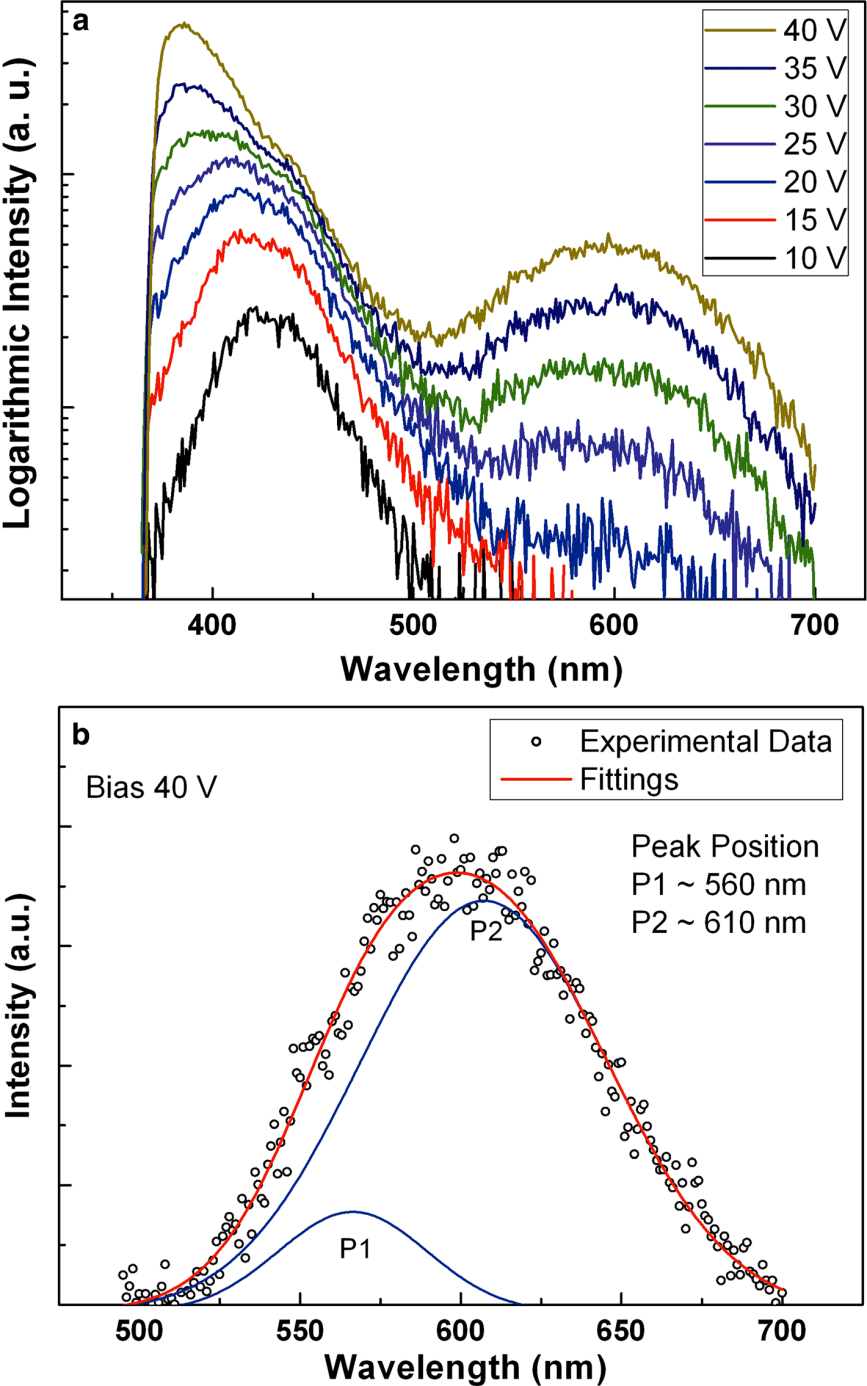
**EL spectra of ZnO/InGaN/GaN heterojunction LED under forward various biases (a) and multi-peak Gaussian fitting (b).** The fitting are from experimental data at the range of 500 to 700 nm.

The EL spectra of ZnO/InGaN/GaN heterojunction LED under various forward biases are shown in Figure 3a. The EL spectra were collected from the back face of the structure at room temperature. As shown in Figure 3a, with a forward bias of 10 V, a blue emission located at 430 nm was observed. Compared with the PL spectra, it can be easily identified that it originated from a recombination in the p-GaN layer. With bias increase, the blue emission peak shifted toward a short wavelength (blueshift). Note that mobility of electrons is faster than holes. Therefore, with low bias, electrons were injected from the n-ZnO side, through the InGaN layer, to the p-GaN side, and little recombination occurred in the n-ZnO and InGaN layers. With bias increase, some holes can inject to the n-ZnO side. Hence, the intensity of emission from the ZnO increased, and as a result, the blue emission peak shifted toward a short wavelength. Additionally, with the bias increase, a peak centered at 600 nm was observed, as shown in Figure 3a. Compared with the PL spectra, the peak is not consistent with p-GaN, ZnO, and InGaN:Si. The peak under the bias of 40 V is thus fitted with two peaks by Gaussian fitting (Figure 3b). The positions of two peaks are 560 and 610 nm, respectively. The emission peak at 560 nm matches well with the PL spectrum of InGaN:Si. However, the emission peak at 610 nm cannot be found in the PL spectra. The PL emission of intrinsic GaN was at 360 nm, and GaN:Mg changes to 430 nm due to transmission from the conduction band and/or shallow donors to the Mg acceptor doping level. Hence, the peak centered at 610 nm might be from the Mg-doped InGaN layer [17].

**Figure 4:**
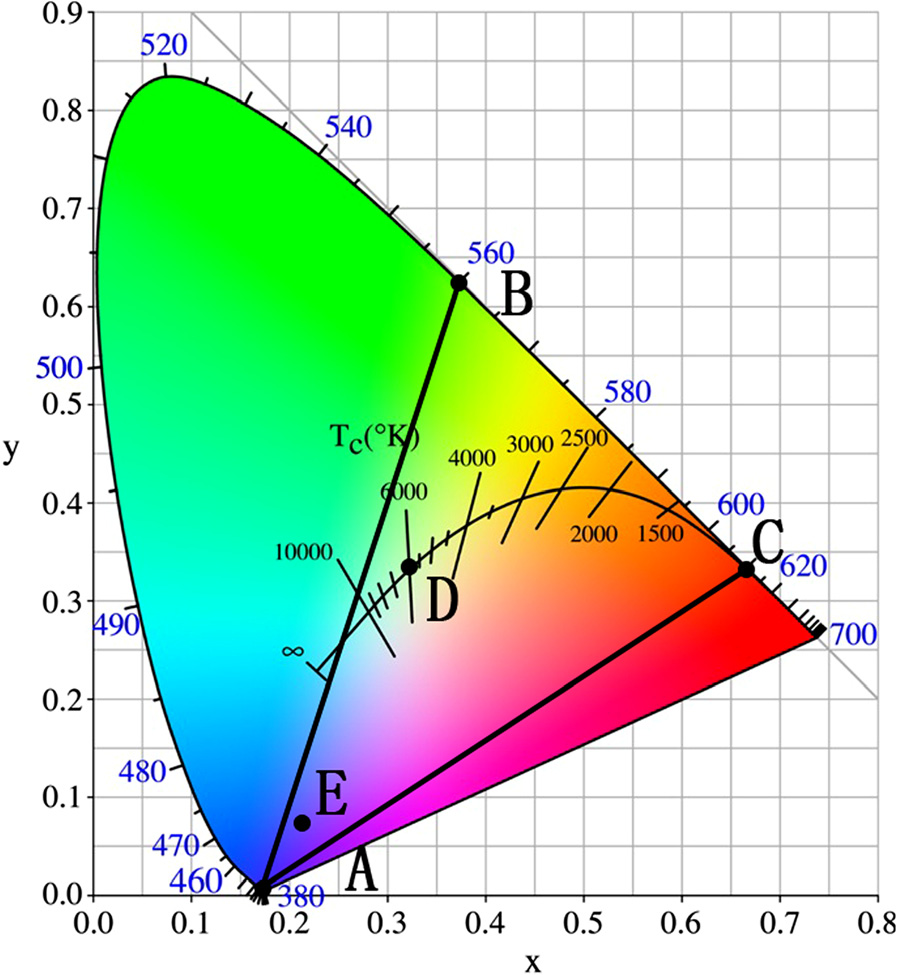
**CIE *x *and *y *chromaticity diagram.**

Figure 4 illustrates the possibility of white light from the ZnO/InGaN/GaN heterostructured LEDs by the Commission International de l'Eclairage (CIE) *x* and *y* chromaticity diagram. Point D is the equality energy white point, and its CIE chromaticity coordinate is (0.33, 0.33). Because the points from 380 to 420 nm on CIE chromaticity diagram are very close, point A is used to represent the blue emission from p-GaN and ZnO. Points B and C represent emissions from InGaN:Si and InGaN:Mg, respectively. As shown in Figure 4, triangle ABC included the ‘white region’ defined by application standards. Therefore, theoretically speaking, the white light can be generated from the ZnO/InGaN/GaN LED with the appropriate emission intensity ratio of ZnO, InGaN:Si, InGaN:Mg, and p-GaN. Therefore, when the EL intensity ratio (380:560:610 nm) was adjusted to 4:5:1, white light was observed from the LED, and its CIE chromaticity coordinate was (0.33, 0.33). Calculating the EL spectrum under the bias of 40 V, the EL intensity ratio (380:560:610 nm) was about 36:1:4, and point E represented emission of the LED. Hence, in order to fabricate WLEDs, the EL intensity of InGaN should be enhanced. In other words, the internal quantum efficiency of the InGaN layers should be improved. Improving the crystalline quality and increasing the carrier concentration of the p-InGaN and n-InGaN layers are the efficient ways to achieve higher internal quantum efficiency.

**Figure 5:**
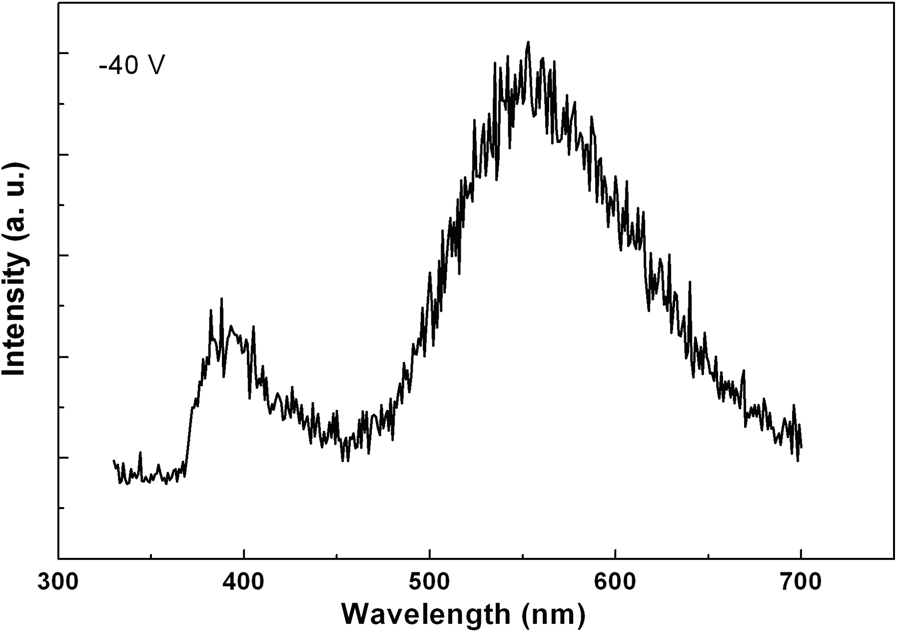
EL spectrum of the ZnO/InGaN/GaN heterojunction LED under the reverse bias.

Furthermore, the EL spectrum under a reverse bias of 40 V is presented in Figure 5. It is much different from that under the forward biases. The EL spectra show a blue emission accompanied by a broad peak centered at 600 nm under forward biases, whereas two emissions (380 and 560 nm) appeared under reverse bias. Obviously, they are attributed to ZnO and InGaN:Si, respectively. The EL mechanism under reverse bias probably is the impact excitation [18].

## Conclusions

In conclusion, we have fabricated heterostructured ZnO/InGaN/GaN LEDs. The EL spectra under forward biases show a blue emission accompanied by a broad peak centered at 600 nm. The peak at 600 nm was deemed to be the combination of the emissions from Si-doped InGaN at 560 nm and Mg-doped InGaN at 610 nm. Counted with the CIE chromaticity diagram, white light can be observed in theory through the adjustment of the emission intensity ratio. Furthermore, a UV emission and an emission peak centered at 560 nm were observed under reverse bias. This work provides a simple way using the emission from ZnO, Mg-doped InGaN, Si-doped InGaN, and p-GaN to obtain white light in theory. With the appropriate emission intensity ratio, ZnO/InGaN/GaN heterostructured LEDs have potential application in WLEDs.

## Competing interests

The authors declare that they have no competing interests.

## Authors’ contributions

TW fabricated the ZnO thin films, performed the measurements of heterostructures, and wrote the manuscript. HW analyzed the results and wrote the manuscript. ZW fabricated the InGaN thin films. CC helped to grow and measure the heterostructures. CL supervised the overall study. All authors read and approved the final manuscript.
